# Effects of Scanning Strategy and Printing Temperature on the Compressive Behaviors of 3D Printed Polyamide-Based Composites

**DOI:** 10.3390/polym12081783

**Published:** 2020-08-10

**Authors:** Jin Wang, Jiangyang Xiang, Hao Lin, Kui Wang, Song Yao, Yong Peng, Yanni Rao

**Affiliations:** 1 Key Laboratory of Traffic Safety on Track of Ministry of Education, School of Traffic & Transportation Engineering, Central South University, Changsha 410075, China; jin.wang@csu.edu.cn (J.W.); 1109160614@csu.edu.cn (J.X.); linhao0613@csu.edu.cn (H.L.); kui.wang@csu.edu.cn (K.W.); song_yao@csu.edu.cn (S.Y.); yong_peng@csu.edu.cn (Y.P.); 2 Joint International Research Laboratory of Key Technology for Rail Traffic Safety, Central South University, Changsha 410075, China; 3 National & Local Joint Engineering Research Center of Safety Technology for Rail Vehicle, Central South University, Changsha 410075, China

**Keywords:** additive manufacturing, fused deposition modeling, scanning strategy, crush behavior, printing temperature

## Abstract

In this work, the effects of scanning strategies and printing temperature on mechanical properties and crush behaviors of columns manufactured using the fused deposition modeling (FDM) technique were studied. The results showed that scanning strategy and printing temperature had significant influences on mechanical response and deformation mode of the columns. The columns printed in different scanning strategies showed significant anisotropy due to the preferred orientation of short fibers during the printing process. The columns printed in a circular direction presented the highest compressive force response. The columns printed with carbon fiber-reinforced polyamide in a circular direction showed the final oblique fracture failure mode, in which there were fiber pull-out and matrix pull-apart on fracture surfaces. Different indicators were also used to evaluate the mechanical properties and crushing characteristics of the columns. The carbon fiber reinforcement columns presented the highest energy absorption, and the glass fiber reinforcement columns showed the highest elastic modulus and yield strength. The results indicated that the scanning strategy and printing temperature not only influenced the elastic modulus and yield strength, but also affected the energy absorption performances of the columns.

## 1. Introduction

Composite materials and structures have drawn increasing attention due to their corrosion resistance, high specific mechanical performance, and lightweight [1,2,3]. There is a growing number of industrial components and structures made by polymers or fiber-reinforced polymer-based composites [4,5]. However, traditional injection molding [6] and hot press molding [7] for polymers limits the manufacture of complicated structures. Therefore, additive manufacturing (AM) as a fast-growing rapid prototyping technique was invented to reach higher efficiency in building complex functional parts. Fused filament fabrication (FFF), which is also well known as fused deposition modeling (FDM), is regarded as a powerful layered manufacturing process due to its flexibility, high material usage, and environmental friendliness [8,9,10]. In recent years, it has been adopted to fabricate structural parts with complicated geometrical shapes using various engineering thermoplastics, such as polyamide (PA) [11,12], polylactic acid (PLA) [13,14], acrylonitrile butadiene styrene (ABS) [15,16], and polyetheretherketone (PEEK) [17,18]. Moreover, it is revealed that fiber reinforcement has great potential in improving the mechanical properties of printed composites. Tekinalp et al. [19] found that the addition of rigid short carbon fiber (CFs) could improve the tensile strength of ABS-based composites. Ferreira et al. [20] investigated the tensile stiffness of the PLA-based composites filled with CFs. It was found that the stiffness increased with increasing CFs content. In our previous studies, we synergistically reinforced the ABS-based printed composites by both short carbon and Kevlar fibers (KFs) in order to tailor the rigidity and ductility [21].

In addition, it is also reported that the mechanical properties of components printed by FDM technique were influenced by printing strategies and parameters. Due to the layered printing method, the components with different building directions have quite distinct mechanical responses. The process parameters may affect the printability and mechanical properties of the materials due to the change of the rheological properties of deposited lines. A considerable amount of literature has been published discussing the properties influenced by the printing process. Gomez-Gras et al. [22] studied the mechanical responses of PLA prepared via the FDM approach. They found that the mechanical properties of the PLA composites were sensitive to layer height, fill density, and nozzle diameter. Chacóna et al. [23] analyzed the influence of layer thickness on the mechanical performance of a PLA. They reported that the strength and stiffness of laminated PLA increased with increasing layer thickness. In their study [24], they also analyzed the effect of build orientation on the mechanical performance of 3D printed continuous fiber-reinforced composites. They found that the flat samples exhibited higher values of strength and stiffness than on-edge samples. Caminero et al. [25] investigated the impact strength of 3D printed continuous fiber-reinforced nylon composites by carrying out the Charpy test. The results showed that the impact strength increased as layer thickness increased in flat samples but, conversely, it decreased in on-edge samples.

Nevertheless, to the best knowledge of the authors, most of the previous studies focused on the tensile and flexural responses of the printed laminated polymer composites. Far too little attention has been paid to the crushing behavior, including energy absorption capability and failure features of fiber-reinforced polymer composites. Moreover, there are few papers reporting the effect of the printing parameters on their compressive mechanical properties. However, composite materials and structures have huge potential to be of novel lightweight structures and energy absorbers with designed and optimized mechanical properties.

Motivated by promising findings and to bridge the knowledge gap, in this work, we presented a series of column-shaped structures made by polyamide and short-fiber-reinforced polyamides fabricated by the FDM process. This paper mainly aimed at the crushing behavior under axial quasi-static compression for the columns printed in three scanning strategies. The materials and processing of columns were described in Section 2. In this section, it was given that the details of morphological characterization studies, thermogravimetric analysis, melt flow index investigation, and quasi-static experimental procedure. In Section 3, the mechanical and crushing properties were evaluated by five essential indicators for columns. The effect of scanning strategy, fiber reinforcement, and printing temperature were analyzed and studied.

## 2. Experimental Procedure

### 2.1. Materials and Processing

**Figure 1:**
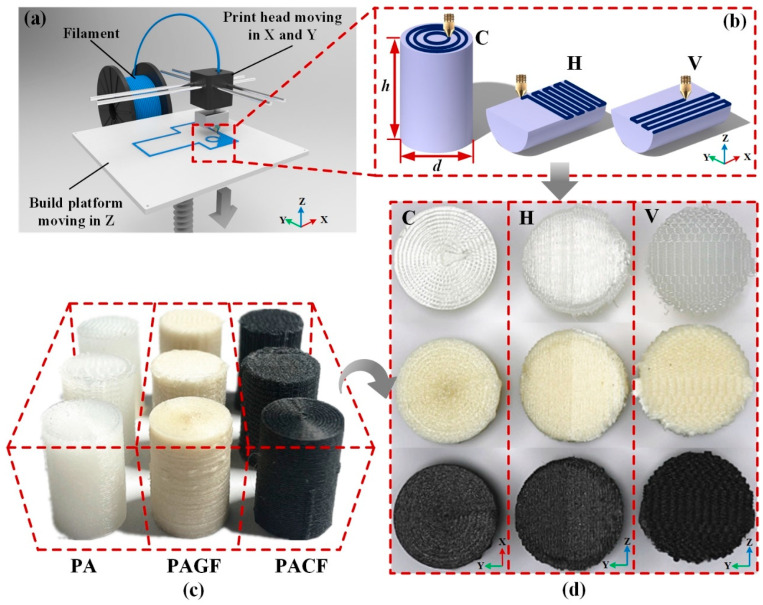
(**a**) Schematic diagram the fused deposition modeling (FDM) technique; (**b**) different scanning strategies in circular, horizontal, and vertical directions, (**c**) isometric view and (**d**) top view of specimens printed by FDM technique in three scanning strategies and three kinds of materials.

In the present work, polyamide (PA), short glass fiber-reinforced polyamide (PAGF), and short carbon fiber-reinforced polyamide (PACF) purchased from eSUN (Shenzhen, China) were used as the printing filaments. The mechanical properties of different materials are shown in Table 1. The geometric models of columns for compression experiments are shown in Figure 1b. The height and diameter are *h* = 15 mm and *d* = 10 mm, respectively. To manufacture the columns by the FDM technique, a 3D printer (Raise3D N2 Plus, Raise3D INC., Costa Mesa, CA, USA) was used.

Figure 1a illustrates the principle of the FDM technique. Filament, with a standard diameter of 1.75mm, was fed by a roller system and melted by a heated die. Then the melted filament was extruded from a nozzle with a diameter of 0.4 mm at different printing temperatures ranging from 240 to 260 °C. The infill percentage was 100%, and the temperature of the build platform was 70 °C. The printing speed was 70 mm/s, and the layer height was 0.15 mm. In this study, three different scanning strategies were selected to print columns shown in Figure 1b, which were circular (C), horizontal (H), and vertical (V). Hence, to name all of the columns, for instance, columns printed by the material of PA in scanning strategies of circular, horizontal, and vertical can be represented as PA-C, PA-H, and PA-V.

### 2.2. Morphological Characterization

The morphological characterization of the printed specimen was obtained by a scanning electron microscopy (SEM, Quanta 650, FEI Co., Hillsboro, OR, USA) with an accelerating voltage of 10 kV. The samples were prepared by the cryofractured method using liquid nitrogen. It can be used to analyze the microstructure of composite materials (raw filament and printed material) including the matrix and the distribution of the short reinforced fibers. Moreover, under different magnifications, interface and interaction of different phases, and failure mechanism of columns with different materials and scanning strategies can be investigated.

### 2.3. Thermogravimetric Analysis (TGA)

Thermogravimetric analysis (TGA) [26] of the materials was carried out using a thermal analyzer (TGA/DSC 3+, Mettler Toledo, Schwerzenbach, Switzerland). Samples, cut from raw filament tows with a mass of 3 ± 1.5 mg, were heated in alumina crucibles. The temperature program for tests consisted of a conditioning period, with the sample maintained at 40 °C for 25 min, followed by linear heating to 700 °C under a nitrogen flow of 20 mL/min. The tests were conducted at a nominal heating rate of 10 °C/min. The results including weight losses versus temperature and decomposition temperatures of the materials were recorded using the STARe software (Mettler Toledo, Schwerzenbach, Switzerland).

### 2.4. Melt Flow Index (MFI)

**Figure 2:**
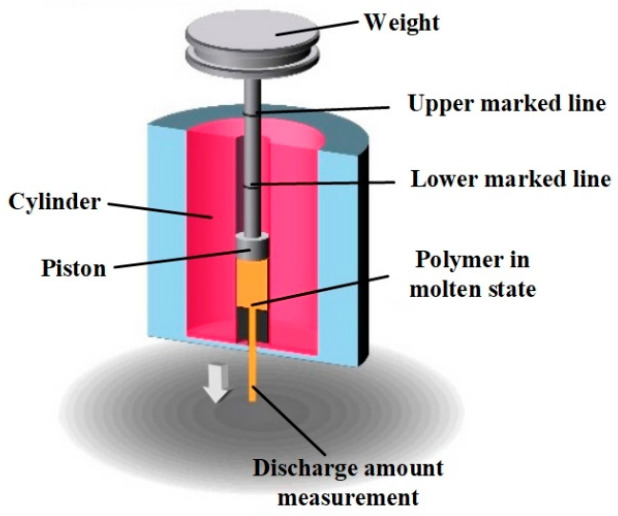
Illustration for the measurement of the melt flow index (MFI) method.

To study the effect of printing temperature on filament, the investigation of the melt flow index (MFI) [27] was carried out, as illustrated in Figure 2. A piston attached to a metal weigh was used to propel filament (20 mm in length) through the nozzle in the molten state at a given temperature. The time was recorded when the piston started moving from the lower-marked line then reached the upper-marked line. The molten filament flowing out of the nozzle was weighed in the recorded time. The mass of the materials extruded per second was regarded as the MFI. In this study, the MFI measurements for studied materials (PA, PACF, and PAGF) were investigated at three temperatures of 240 °C, 250 °C, and 260 °C. To reduce the influence of the experimental error, three repetitive testings were carried out.

### 2.5. Quasi-Static Axial Compression

**Figure 3:**
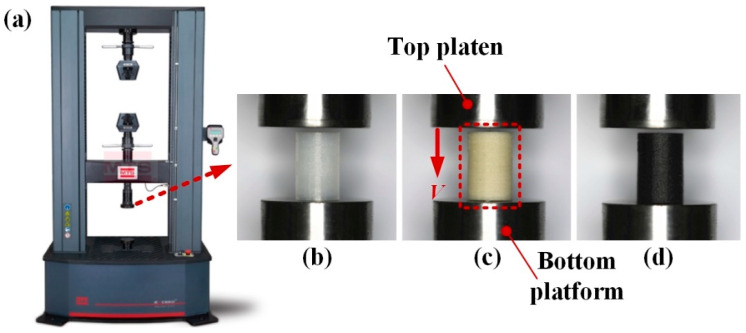
Quasi-static compression experiments and specimens printed by different materials: (**a**) universal material testing machine; (**b**) polyamide (PA); (**c**) short glass fiber-reinforced polyamide (PAGF); (**d**) short carbon fiber-reinforced polyamide (PACF).

To study and analyze the force response and crushing behaviors of columns, quasi-static compressive tests were carried out by using a universal material-testing machine (E44, MTS Systems Co., Eden Prairie, MN, USA) at room temperature, as shown in Figure 3a. The specimens were placed at a rigid platform, while the load was applied by another rigid platen moving down at a constant velocity of 2 mm/min. Three repetitive tests for each condition were carried out. The corresponded load was recorded within the crushing displacement of 10 mm, which was 2/3 of the original length [28,29]. The whole collapse process was captured by a digital camera (EOS 5D Mark IV, Canon Inc., Tokyo, Japan), and the force-displacement data were collected by a data acquisition system of the MTS.

To quantitatively evaluate the crushing behaviors of the columns, beside Young’s modulus and yield stress, several indicators were also used in this study as follows [30,31,32]. Energy absorption (EA) is total energy absorption during the crushing process, which is calculated as the area under a crushing force-displacement curve,

(1) $$\mathrm{EA}=\int_{0}^{d}F(x)dx$$

where *d* denotes the axial crushing displacement, and *F*(*x*) is the crushing force. 

Specific energy absorption (SEA) is the energy absorption per unit mass of an energy absorber, which is believed as the most critical indicator in designing an energy absorption device. SEA can be formulated by EA as,

(2) $$\mathrm{SEA}=\frac{\mathrm{EA}}{m}$$

where *m* is the total mass of the specimen.

Mean crushing force (MCF) is the average value of crushing force during the whole crushing process, which is obtained by dividing the total absorbed kinetic energy by the crushing distance *d*, described as follows:

(3) $$\mathrm{MCF}=\frac{\mathrm{EA}}{d}$$

## 3. Results and Discussion

### 3.1. Morphological, Thermal and Rheological Behaviors of Raw Filament

**Figure 4:**
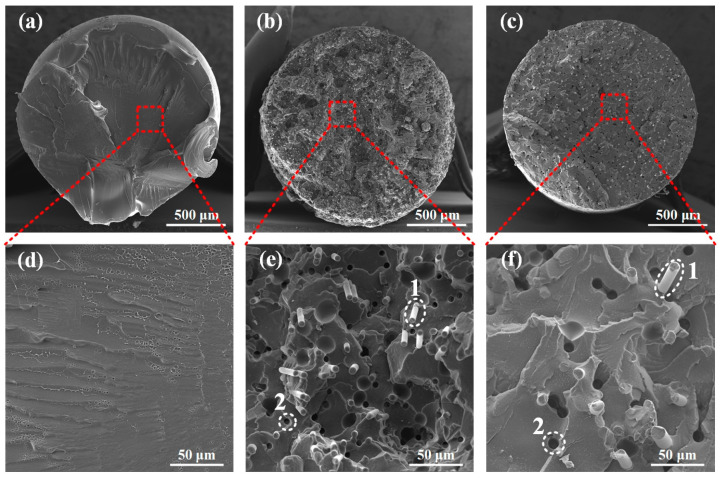
SEM images of printing filaments for (**a**) PA, (**b**) PACF, (**c**) PAGF, and the corresponding enlarged figures (**d**–**f**) (dashed circles 1 indicate the short fibers and dashed circles 2 indicate dark voids caused by the pulling out of the short fibers).

To study the microstructure and fiber distribution within the matrix for PA, PACF, and PAGF, scanning electron microscopy (SEM) observations were applied. The cryofractured cross-sectional morphologies of the raw filament are shown in Figure 4. It can be clearly seen that in both PACF and PAGF, short fibers (dashed circle 1) randomly and well-embedded in the PA matrix. The short fibers were generally in the axial orientation of filament but in a random slant angle. It can be measured that the average diameter of the short carbon fiber was 4.38 ± 0.5 μm, while that of the short glass fiber was 7.88 ± 0.5 μm. Besides, dark voids caused by the pulling out of the short fibers during the cryofractured process were observed in the enlarged view (Figure 4e,f). 

**Figure 5:**
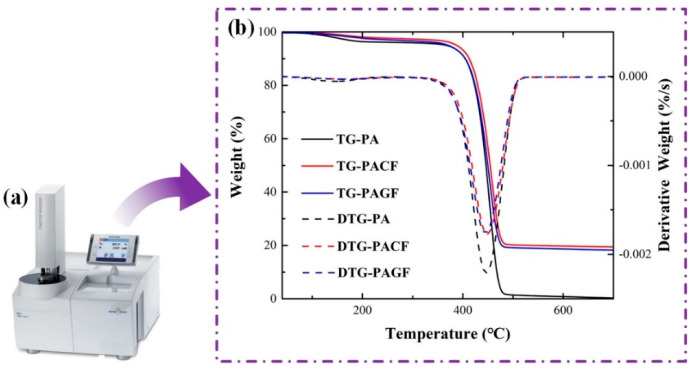
Thermogravimetric analysis (TGA): (**a**) Mettler TGA/DSC 3+; (**b**) results.

To investigate the weight fraction of the fibers, TGA was carried out by a thermal analyzer (TGA/DSC 3+, Mettler Toledo, Schwerzenbach, Switzerland). The TGA results are shown in Figure 5. In this figure, the curves of weight losses (%) versus temperature (TG) and derivative weight losses (%/s) versus temperature (DTG) for PA, PACF, and PAGF printing filaments are exhibited. It can be observed that the weight of all three printing filaments lost slightly from 40 °C to 420 °C. This was due to the evaporation of moisture for the printing filaments within this stage. With the increase of temperature, the weight of three filaments dropped sharply until 470 °C, with the highest rate of derivate weight value at 450 °C. The main reason for the sharp drop weight of the materials within this range of temperatures was the decomposition of the PA matrix. After this stage, the weight for all the samples were lost slowly until 700 °C in our investigated range. More specifically, the weight of PA decreased to close to zero at the end of the investigation, indicating the full decomposition of the PA matrix. For the composite materials of PACF and PAGF, it was well known that the decomposition temperatures of carbon and glass fibers are usually much higher than 1000 °C [26]. Thus, the residual weight should be attributed to the presence of carbon fibers and glass fibers. Referring to the value in Figure 5b, it can be concluded that glass fiber and the carbon fiber contents in PACF filaments and PAGF filaments were 19.8 ± 0.3 wt. % and 19.7 ± 0.8 wt. %, respectively.

**Figure 6:**
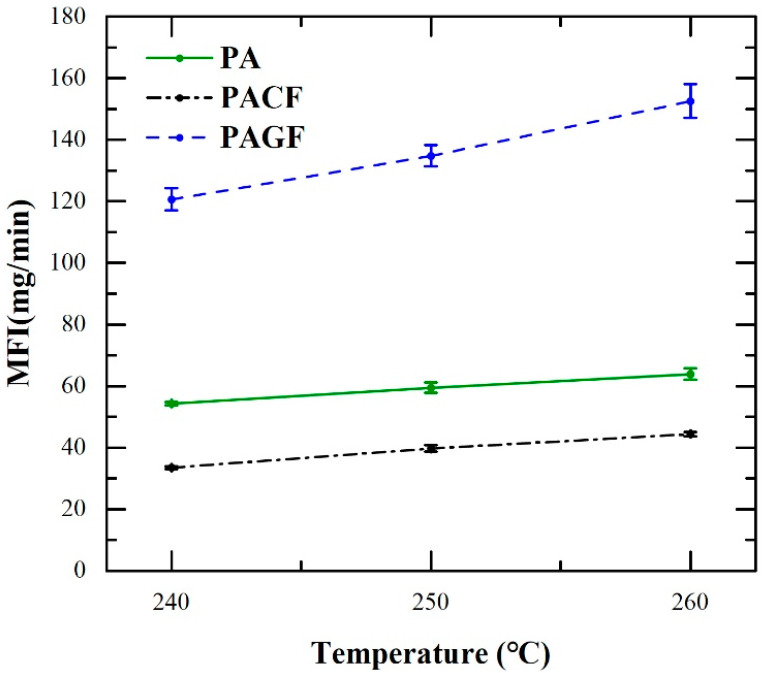
Melt flow index (MFI) of PA, PAGF, and PACF at different temperatures.

The MFI of the printing filament can be cited as one of the most critical indicators related to the FDM printing process [33,34,35]. It is highly correlated with the thermal viscosity of the filament at a specific printing temperature [27]. Figure 6 shows the results of MFI for all three printing materials. Referring to the error bar, MFI of all three filaments exhibited good repeatability. Moreover, PAGF showed the highest MFI values, while the MFI values of PACF were the lowest for the three studied temperatures. All three materials presented an increasing tendency with increasing temperature. It could be concluded that the higher the temperature, the higher the melt flow of printing materials. Compared with PA and PACF, PAGF showed the highest rising tangent, indicating that the rheological behavior of PAGF was more sensitive to temperature. 

### 3.2. Morphological Properties of Printed Composites

**Figure 7:**
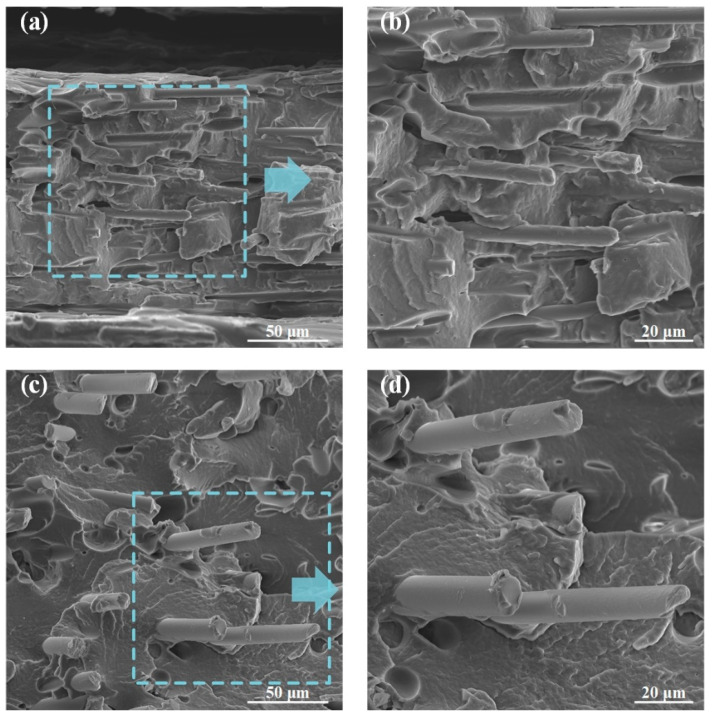
SEM images of printed (**a**,**b**) PACF and (**c**,**d**) PAGF composites built in a horizontal direction.

Figure 7 presents the cryofractured cross-sectional morphologies of PAGF and PACF built in horizontal direction and at 250 °C. The morphological properties of the specimen built in other directions and at other temperatures were not shown here because their morphological behaviors were similar. Compared with the preferred orientation of short fibers in printing filament (see Figure 4), the fibers in printed samples were significantly combed along the line deposition direction, attributing to the smaller nozzle diameter of the printer rather than the filament extruder one. The highly orientated fibers demonstrated that the melt flow of the materials during the printing process could have an important effect on the morphological characteristics. Therefore, the mechanical properties of columns would highly depend on the scanning strategy of the printing process due to the anisotropy of materials.

### 3.3. Crushing Behavior of 3D Printed Columns

**Figure 8:**
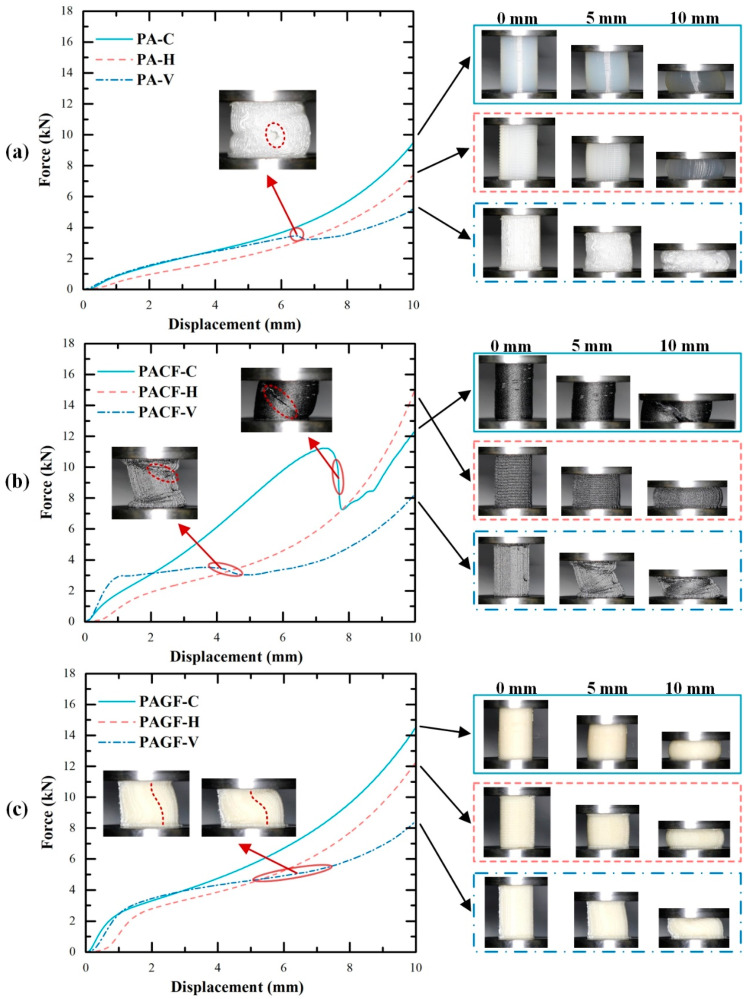
Force responses and corresponding deformation and collapse modes of columns: (**a**) PA; (**b**) PACF; (**c**) PAGF (printed at 240 °C) (red dashed circles and lines drawn in pictures indicate the collapse mode of columns).

Figure 8 gives the quasi-static axial compression processes and corresponding force-displacement curves for three materials built in three directions. It was seen that different scanning strategies resulted in different deformation modes of the samples. PA-C and PA-H columns presented symmetric radial expansion, while the PA-V columns showed symmetric folding. During the compression process of PA-V, a crack occurred (see the red dashed circle in Figure 8a) leading a drop stage of compressive force. The main reason was that symmetric folding mode resulted in the filament apart when folding force exceeded the bonding force between print filaments. The corresponding force-displacement curves can be divided into three stages. At the beginning of the compression process, the columns first experienced the elastic stage, where the force increased linearly with increasing compression displacement. Then the curves smoothly transited to the plastic stage, and the significant deformation can be observed at the meantime. The force kept increasing but with a relatively low gradient compared with that in elastic stage. When columns compressed to the densification point, they finally entered the densification stage. It was regarded as an ineffective stage for energy absorption during which the force increased rapidly in a short displacement. 

Compared with PA columns, the fiber-reinforced columns (PACF and PAGF) built in all three directions showed the much higher force response. Besides, the columns in the circular scanning strategy showed the highest compressive force. It was also worth paying attention that collapse modes of PACF and PAGF columns were also different from PA columns due to the reinforcement of the short fibers. More specifically, PACF-C showed oblique fracture failure modes accompanied by a rapid drop of the compressive force (see Figure 8b). The main reason was that the printed circular filament showed tensile deformation when columns were compressed to expansion. Because of the intrinsic brittle property of the short carbon fibers, PACF-C presented the deformation mode in a brittle manner. It was well known that during the compressive process of the brittle materials, the 45° plane was of the highest shear stress so that oblique fracture occurred when it exceeded the ultimate stress of the materials.

**Figure 9:**
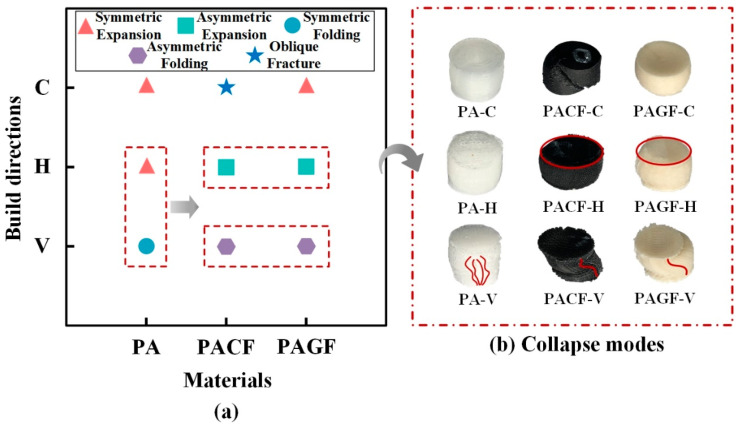
(**a**) The distribution of collapse modes for different materials built at different directions; (**b**) captures of collapse modes of columns (red ellipses indicate symmetric expansion modes and red lines indicate the folding modes).

Moreover, due to the existence of highly orientated (shown in Figure 7) short fibers, the elastic modulus of the materials along the printing direction was reinforced by fibers. Thus, the axial elongation of the filament was much less than that of radial elongation. Therefore, fiber-reinforced columns in the horizontal direction (PACF-H and PAGF-H) presented asymmetric expansion, while PA columns in horizontal direction showed symmetric expansion (also see Figure 9). Besides, compared with the symmetric folding of the PA-V, PACF and PAGF presented the asymmetric folding, and there was no delamination observed during the compression. This demonstrated that there was a stronger interaction between fiber-reinforced printed filaments.

Overall, as were shown in Figure 9, there were five different collapse modes among PA, PACF, and PAGF printed in three scanning strategies: symmetric expansion, asymmetric expansion, symmetric folding, asymmetric folding, and oblique fracture. The short fibers not only enhanced the compressive force but also influenced the deformation and failure modes of the columns. The deformation mode of columns during the compressive process determined the final collapse mode. The scanning strategy played an essential role in the compressive behavior of 3D printed columns. 

#### 3.3.1. Effect of Scanning Strategy

**Figure 10:**
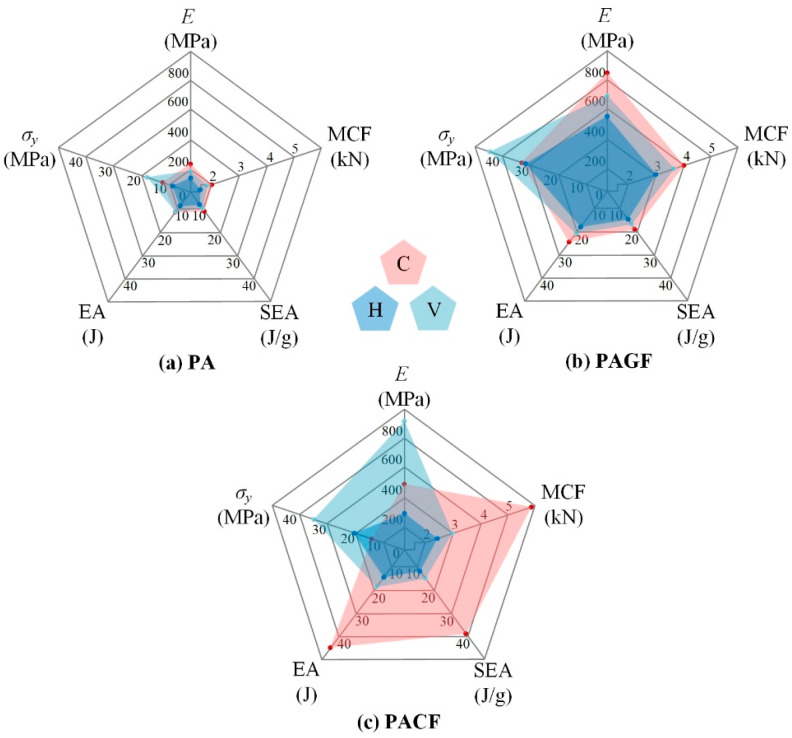
Assessment of printed columns based on indicators of crushing properties: (**a**) PA; (**b**) PAGF; (**c**) PACF (printed in three directions and printed at 240 °C).

To further evaluate the effect of scanning strategy on crushing properties of 3D printed columns, the elastic modulus, yield strength, EA, SEA, and MCF were quantitatively compared. The assessment results were as shown in Figure 10. Among all columns, horizontal direction showed the lowest values for all indicators (except for the yield stress of PACF-H (a little bit higher than that of PACF-C)). The PAGF columns printed in all three scanning strategies presented superior mechanical properties but relatively weak in energy absorption. The effect of scanning strategy was most significant among PACF columns. Because of the short fiber reinforcement and its orientation along the filament, the PACF columns in vertical scanning strategy showed the highest elastic modulus and yield stress. The PACF columns in circular had advantages on energy absorption over columns in horizontal and vertical scanning strategies but showed the low elastic modulus and yield stress. It can be regarded as a potential energy absorber due to the high SEA with a low peak crushing force [36,37]. Referring to Figure 8 and Figure 9, PACF columns in a circular direction showed the highest compressive force level accompanied by an oblique fracture collapse mode. A lot of energy can be absorbed during the fracture of materials.

**Figure 11:**
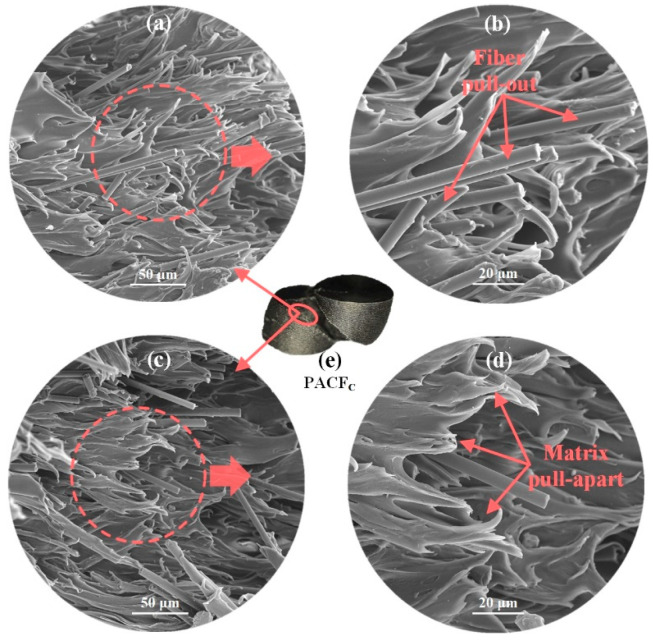
(**a**) and (**c**): SEM images of fracture surfaces of PACF-C; (**b**) and (**d**): enlarged views; (**e**) the final deformation mode of PACF-C.

Furthermore, Figure 11 gives SEM images of fracture surfaces for PACF-C. The carbon fiber pull-out was obviously observed, which was considered as an essential mode for energy absorption. In addition, the matrix pull-apart presented in fuzzy morphology was found on fractured surfaces. This demonstrated that the PA matrix had a ductile tensile failure when it was pulled apart during the fracture process, which can also result in a mass of energy absorption. Therefore, the PACF columns printed in the circular direction, which was the only column that occurred oblique fracture, had the highest energy absorption.

#### 3.3.2. Effect of Short Fiber Reinforcement on Energy Absorption

**Figure 12:**
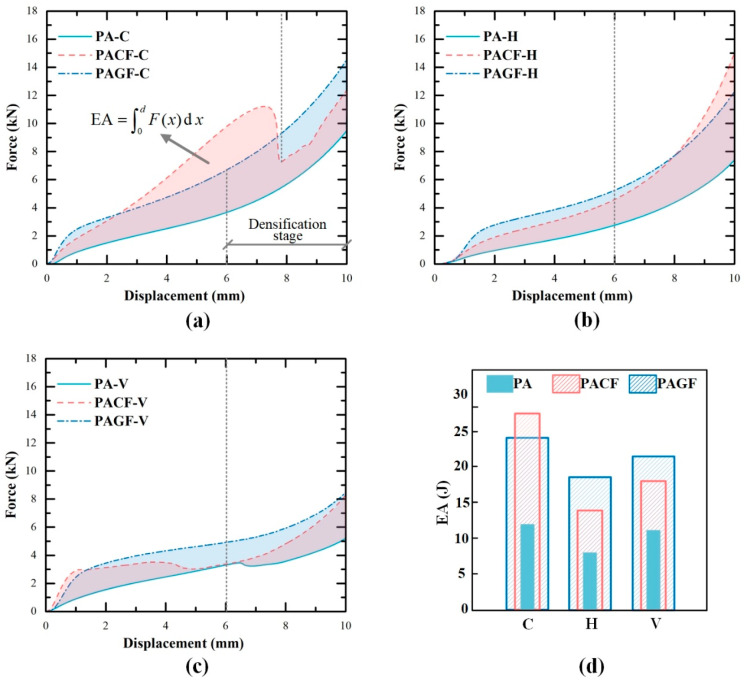
Force-displacement curves of different columns in different scanning strategies: (**a**) circular; (**b**) horizontal; (**c**) vertical; and (**d**) energy absorption capabilities of different columns.

Figure 12 shows the force-displacement curves of PA and short fiber-reinforced PA columns. The shaded area represented the improved energy absorption for PACF and PAGF compared to PA columns. To analyze the improvement of short fibers, the effective energy absorption of columns at the compressive displacement of 6 mm (before densification stage) was compared. Although the densification point of PACF-C was delayed due to the special fracture collapse mode, energy absorption at the displacement of 6 mm was still used for comparison. The specific EA of columns were shown in Figure 12d. It was seen that the addition of the short fibers significantly improved the energy absorption for columns printed in all three scanning strategies. The short carbon fiber presented more significant improvement than the glass fiber for columns printed in the circular direction. In contrast, the glass fiber showed better enhancement effect on the energy absorption of columns in horizontal and vertical directions. This indicated that improvement in energy absorption for columns in different print directions was highly dependent on the type of short fiber. The carbon fiber reinforcement presented the highest enhancement on energy absorption of 132.3% compared with the PA column. The glass fiber showed the most significant improvement of 128.8% for the columns printed horizontal direction. 

#### 3.3.3. Effect of Printing Temperature

**Figure 13:**
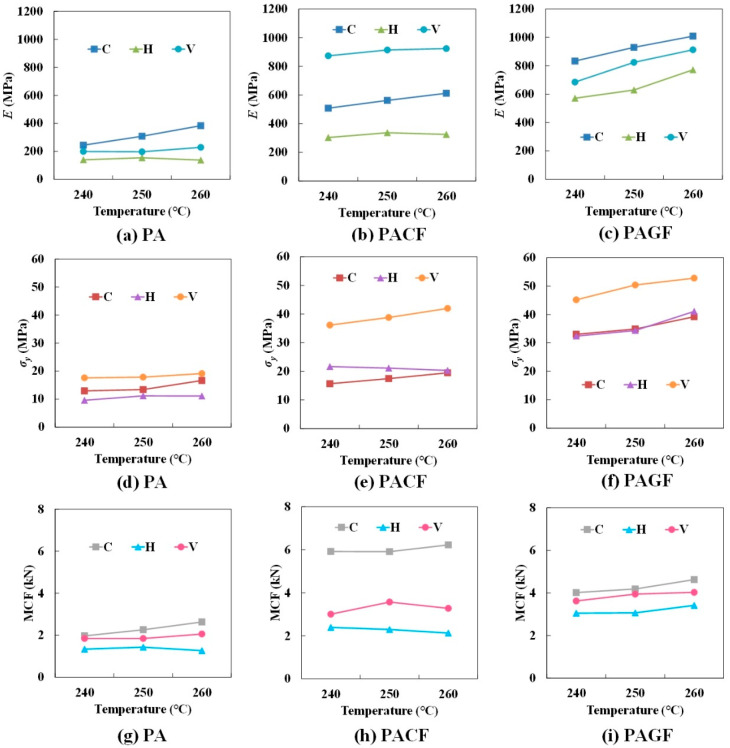
Effect of printing temperature on elastic modulus of: (**a**) PA; (**b**) PACF; (**c**) PAGF; and on yield strength of: (**d**) PA; (**e**) PACF; (**f**) PAGF; and MCF of: (**g**) PA; (**h**) PACF; (**i**) PAGF.

Table 2 presents the specific experimental results of quasi-static axial compression. Figure 13 shows elastic modulus, yield strength, and MCF results (average value of three repetitive tests) of columns printed by three materials in three scanning strategies. With the increase of printing temperature from 240 °C to 260 °C, three indicators showed a generally increasing tendency. Except for the PA and PACF columns in the horizontal direction, the elastic modulus, yield strength, and MCF showed less sensitivity to printing temperature. The main reason was that the increase of the printing temperature resulted in much influence on the melt flow along the filament length. This indicated that appropriately increasing the printing temperature can enhance the mechanical and crushing properties. 

**Figure 14:**
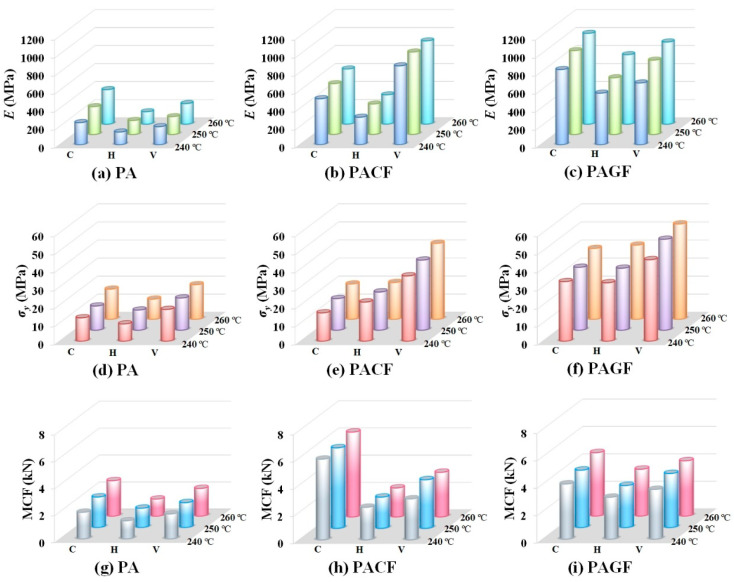
Elastic modulus of columns made of: (**a**) PA; (**b**) PACF; (**c**) PAGF; yield strength of columns made of: (**d**) PA; (**e**) PACF; (**f**) PAGF; and MCF of columns made of: (**g**) PA; (**h**) PACF; (**i**) PAGF.

Overall, as shown in Figure 14, PACF columns in the vertical direction and PAGF columns in the circular direction presented the relative high-elastic modulus. The PAGF columns in vertical direction showed the highest yield stress. The PACF columns in circular had the advantages of mean crushing force. The results demonstrated that PAGF-V and PACF-C had great potential for bearing capacity and energy absorption. 

## 4. Conclusions

In this study, we mainly investigated the effect of scanning strategy, fiber reinforcement, and printing temperature of the FDM technique on compressive behavior for the columns. Based on the results from experimental tests, the following conclusions were drawn:

(1) The morphological investigation indicated a good distribution of short carbon and glass fiber in the PA matrix. From the results of TGA, it was found that raw filament of PACF and PAGF had a short fiber content of 19.8 ± 0.3 wt. % and 19.7 ± 0.8 wt. %, respectively.

(2) The scanning strategy of columns had the significant effect on compressive deformation mode and force response. Five deformation modes of were discovered. The oblique fracture was found in PACF-C, which had the highest energy absorption. The fiber pull-out and matrix pull-apart were found on the fracture surfaces. 

(3) Due to the fiber reinforcement, PAGF columns presented higher bearing capacity, while PACF columns can show the great potential on energy absorption. The highest enhancement of PACF-C on energy absorption reached 132.3%.

(4) Increasing the printing temperature from 240 to 260 °C can show an improvement tendency in the mechanical and crushing properties of columns. The elastic modulus of PAGF columns showed the most significant sensitivity to printing temperature. 

## Figures and Tables

**Table 1:** Material properties of the print filaments.

Material Properties	PA	PAGF	PACF
Density (g/cm^3^)	1.12	1.24	1.35
Tensile strength (MPa)	57	75	101
Elongation (%)	196	26	17
Flexural strength (MPa)	57	122	121

**Table 2:** Experimental results of quasi-static axial compression.

Columns	Print Temperature (°C)	Elastic Modulus (MPa)	Yield Strength (MPa)	MCF (kN)
PA-C	240	244.43 ± 5.73	12.93 ± 0.52	1.97 ± 0.02
	250	308.05 ± 7.64	13.40 ± 0.42	2.26 ± 0.05
	260	383.40 ± 11.84	16.65 ± 0.29	2.63 ± 0.04
PA-H	240	139.76 ± 4.71	9.56 ± 0.34	1.33 ± 0.04
	250	154.12 ± 3.20	11.16 ± 0.21	1.43 ± 0.02
	260	137.79 ± 3.72	11.11 ± 0.11	1.27 ± 0.02
PA-V	240	199.24 ± 7.44	17.60 ± 0.14	1.84 ± 0.07
	250	197.35 ± 13.49	17.84 ± 0.25	1.84 ± 0.07
	260	228.85 ± 3.23	19.10 ± 0.33	2.06 ± 0.03
PACF-C	240	508.37 ± 30.80	15.67 ± 1.52	5.92 ± 0.10
	250	562.66 ± 52.82	17.48 ± 2.14	5.91 ± 0.14
	260	612.25 ± 34.95	19.49 ± 0.22	6.23 ± 0.12
PACF-H	240	303.15 ± 10.15	21.66 ± 0.55	2.39 ± 0.08
	250	336.66 ± 23.52	21.14 ± 1.50	2.29 ± 0.16
	260	324.68 ± 23.19	20.31 ± 0.88	2.13 ± 0.14
PACF-V	240	873.80 ± 22.65	36.16 ± 1.40	3.00 ± 0.04
	250	914.13 ± 23.41	38.81 ± 3.02	3.57 ± 0.07
	260	924.17 ± 47.20	41.98 ± 1.67	3.27 ± 0.13
PAGF-C	240	833.07 ± 95.32	33.02 ± 2.80	4.02 ± 0.15
	250	929.58 ± 30.88	34.87 ± 1.04	4.19 ± 0.02
	260	1008.19 ± 12.97	39.21 ± 0.30	4.63 ± 0.08
PAGF-H	240	571.22 ± 16.88	32.39 ± 0.05	3.05 ± 0.03
	250	628.87 ± 34.75	34.36 ± 1.59	3.07 ± 0.14
	260	771.47 ± 3.28	41.08 ± 0.71	3.42 ± 0.14
PAGF-V	240	684.63 ± 17.90	45.17 ± 1.23	3.62 ± 0.08
	250	824.13 ± 8.81	50.40 ± 0.39	3.94 ± 0.09
	260	912.32 ± 47.95	52.81 ± 1.15	4.03 ± 0.08
